# An Experimentally Validated Finite Element Model of the Lower Limb to Investigate the Efficacy of Blast Mitigation Systems

**DOI:** 10.3389/fbioe.2021.665656

**Published:** 2021-05-28

**Authors:** Eduardo A. Rebelo, Grigoris Grigoriadis, Diagarajen Carpanen, Anthony M. J. Bull, Spyros D. Masouros

**Affiliations:** Department of Bioengineering, Imperial College London, London, United Kingdom

**Keywords:** under-body blast, blast injury, trauma biomechanics, lower limb biomechanics, military combat boots

## Abstract

Improvised explosive devices (IEDs) used in the battlefield cause damage to vehicles and their occupants. The injury burden to the casualties is significant. The biofidelity and practicality of current methods for assessing current protection to reduce the injury severity is limited. In this study, a finite-element (FE) model of the leg was developed and validated in relevant blast-loading conditions, and then used to quantify the level of protection offered by a combat boot. An FE model of the leg of a 35 years old male cadaver was developed. The cadaveric leg was tested physically in a seated posture using a traumatic injury simulator and the results used to calibrate the FE model. The calibrated model predicted hindfoot forces that were in good correlation (using the CORrelation and Analysis or CORA tool) with data from force sensors; the average correlation and analysis rating (according to ISO18571) was 0.842. The boundary conditions of the FE model were then changed to replicate pendulum tests conducted in previous studies which impacted the leg at velocities between 4 and 6.7 m/s. The FE model results of foot compression and peak force at the proximal tibia were within the experimental corridors reported in the studies. A combat boot was then incorporated into the validated computational model. Simulations were run across a range of blast-related loading conditions. The predicted proximal tibia forces and associated risk of injury indicated that the combat boot reduced the injury severity for low severity loading cases with higher times to peak velocity. The reduction in injury risk varied between 6 and 37% for calcaneal minor injuries, and 1 and 54% for calcaneal major injuries. No injury-risk reduction was found for high severity loading cases. The validated FE model of the leg developed here was able to quantify the protection offered by a combat boot to vehicle occupants across a range of blast-related loading conditions. It can now be used as a design and as an assessment tool to quantify the level of blast protection offered by other mitigation technologies.

## Introduction

Recent armed conflicts have been marked by the use of explosive devices (EDs) to attack armored vehicles (Ramasamy et al., 2011). In an under-body blast (UBB) scenario, where an explosive detonates underneath a vehicle, the resulting detonation products from the explosion together with soil particles are propelled upward causing localized deformation of the floor of the vehicle, transferring loading through the floor to the lower extremities of the occupants, resulting in difficult-to-treat injuries (Ramasamy et al., 2011). These high energy injuries result in, among others, intra-articular fractures which pose many challenges and complications to patients and surgeons. Such injuries are associated with high levels of morbidity, high amputation rates, and a low return-to-duty rate (Doukas et al., 2013; Ramasamy et al., 2013).

In order to analyze the injury mechanisms associated with UBB blast injuries, there has been an effort to model UBB conditions. Experimental setups to emulate UBB conditions utilizing postmortem human subjects (PMHS) in the laboratory, albeit invaluable, are expensive, laborious, and can only provide information where sensors are placed. Physical models have been complemented over the years with computational models. Very limited UBB finite-element (FE) models have been developed to explore the injury pathways in more detail, mostly with representations of anthropometric test devices (Newell et al., 2016; Baker et al., 2017, 2018), and rarely of the relevant human anatomy structures (Dong et al., 2013). All these FE models have been compared against load cases from one experimental set up only, rendering their utility limited; they do not include an assessment of the model’s response in additional set-ups to span a representative range of UBB loading. Furthermore, in order to mitigate severe lower limb injuries, the effort of predicting lower limb injury with these models needs to be complemented with the development of tools to assess the efficacy of protective systems. The aim of this study is the development and validation of an FE model of the leg and to provide an exemplar of its potential utility to assess countermeasures.

## Materials and Methods

A cadaveric lower limb (male, 35 years old) was used for physical and FE modeling of UBB conditions. The weight and height of the specimen closely matched the dimensions of a 50th percentile American male (height: 173 m, mass: 72.6 kg). The Tissue Management Committee of the Imperial College Tissue Bank Ethics Committee granted ethical approval for this study (Ethical approval number: 12-WA-0196).

### Experiment

Experimental testing was conducted using an anti-vehicular under-body blast injury simulator (AnUBIS) (Masouros et al., 2013). The loading plate of the simulator accelerates to a defined target velocity before coming rapidly to rest. The configuration of the cadaveric leg during the test was set to represent the alignment of the foot and ankle in a seated posture. This involved setting the foot to 0°of dorsiflexion and the tibia angle with the floor to 90° using a digital inclinometer (model SlopeView TLL-90E, MIB Instruments Co., Hong Kong SAR). The loading plate includes a force sensor (200C20, PCB Piezotronics, Depew, NY, United States) that measures the force transmitted under the heel in the direction of loading. Additionally, the loading plate was instrumented with two uniaxial accelerometers (model 350D02, PCB Piezotronics Ltd., Hitchin, United Kingdom) to monitor the motion of the plate.

The peak impact velocity was computed by integrating the measurement data from the uniaxial accelerometers secured on the loading plate. The signals from the accelerometers were filtered using a CFC 1000 filter. A low-pass second-order Butterworth filter was used to filter force sensor data. All data were recorded at a frequency of 25 kHz using a PXIe data acquisition system (model 1082, National Instruments, Austin, TX, United States) and a custom-written LabVIEW code (v2012, National Instruments, Austin, TX, United States). An anti-aliasing filter was used on accelerometers and strain gauges as part of the PXIe data acquisition system. The test was conducted in an unbooted configuration and in a configuration where a United Kingdom size 10 Meindl Desert Fox combat boot (Lukas Meindl GmbH & Co., Germany) was placed on the foot of the cadaveric leg. A non-injurious test condition was selected to ensure that there were no fractures before the second test in AnUBIS without a combat boot.

The first test was conducted with the combat boot and the subsequent test without a combat boot. The lower limb was scanned after the first test at the foot and at the lower leg with an x-ray fluoroscan (Vertec, Reading, United Kingdom) to assess whether there were any fractures. No fractures were seen. The lower limb was also scanned after the second and final impact test and no fractures were seen. The target peak velocity of the loading plate was 5.2 m/s.

### Finite-Element Model of the Human Leg

The cadaveric leg was scanned using Computed Tomography (CT) (Siemens Somatom Definition AS 64, Erlangen, Germany) and the images were used to build the geometry of the FE model. The CT scan was imported to an image processing software (v2015, Mimics, Materialize) where the anatomical components of the specimen were reconstructed into 3D objects and later exported to an FE pre-processor software (v2014, Altair HyperMesh) for meshing. The thickness estimations for different regions were compared with the data and distribution maps reported in experimental studies (El-Khoury et al., 2004; Millington et al., 2007). Cortical thickness for the tarsal bones was calculated from the available CT scans and compared with the measurements reported in the literature (Pal and Routal, 1998; Sabry et al., 2000).

The trabecular structures of the calcaneus, talus, tibia, fibula, and the plantar soft tissues were modeled with three-dimensional hexahedral and pentahedral elements. The remainder cortical bone and cartilaginous tissues were modeled with Key-Hoff quadrilateral shell elements (PSHELL1) created in HyperMesh using the faces feature. These elements were defined with a bottom reference surface and an outwardly growing thickness. For the calcaneus and talus, a layered approach was pursued to represent cartilage and cortical bone with just one layer of shell elements. After executing equivalence and optimization, the mesh was refined manually. It was found that fewer than 1% of solid elements had a Jacobian less than 0.3 and fewer than 3% shell elements were found to have a Jacobian value less than 0.7. The average element size length was set at 2.5 mm.

The FE model of the leg was set up in Patran (MSC Software, v2018). Material models were selected from previous lower limb FE studies and material characterization experiments (Table 1). A piecewise linear plasticity material model was used to represent cortical bone. Ligaments were represented with one dimensional spring elements able to withstand tension only and defined by force-displacement curves based on previous studies. All simulations were run in MSC.Dytran (v2018, MSC Software).

**TABLE 1:** Material properties used in the FE models.

Anatomical component	Material model	Material parameters
Trabecular bone	Linearly elastic	*ρ = 1.1, E = 0.3, v = 0.3* (McElhaney, 1966)
Cortical bone	Piecewise linear plasticity	ρ = 2, *E*_*quasistatic*_ = 17.5, *E*_*dynamic*_ = 19, *v* = 0.3, σ = 165 (Sommer and Olaf, 1994; McElhaney, 1966; Hansen et al., 2008)
Cartilage	Linearly elastic	*ρ = 1, E = 0.3, v = 0.3* (Kitaoka et al., 1994; Iwamoto et al., 2005; Shin et al., 2012)
Ligaments	Tensile-only non-linear spring	
Heel Fat Pad	Quasi-linear viscoelastic	*ρ* = 0.97, *A* = 0.1, *B* = 1.258, *v* = 0.495 (Grigoriadis et al., 2019) *Q* = 1.6
Mild steel	Linearly elastic	*ρ* = 7.89, *E* = 205, *v* = 0.29 (Masouros et al., 2013)

*E, Young’s modulus [GPa]; v, Poisson’s ratio; ρ, density [g/cm3]; σ, compressive ultimate stress [MPa]; A (C_01_) and B (C_10_), Mooney-Rivlin model parameters [MPa]; Q, Quadratic viscosity parameter.*

### Model Calibration – Simulation of the AnUBIS Setup

**FIGURE 1:**
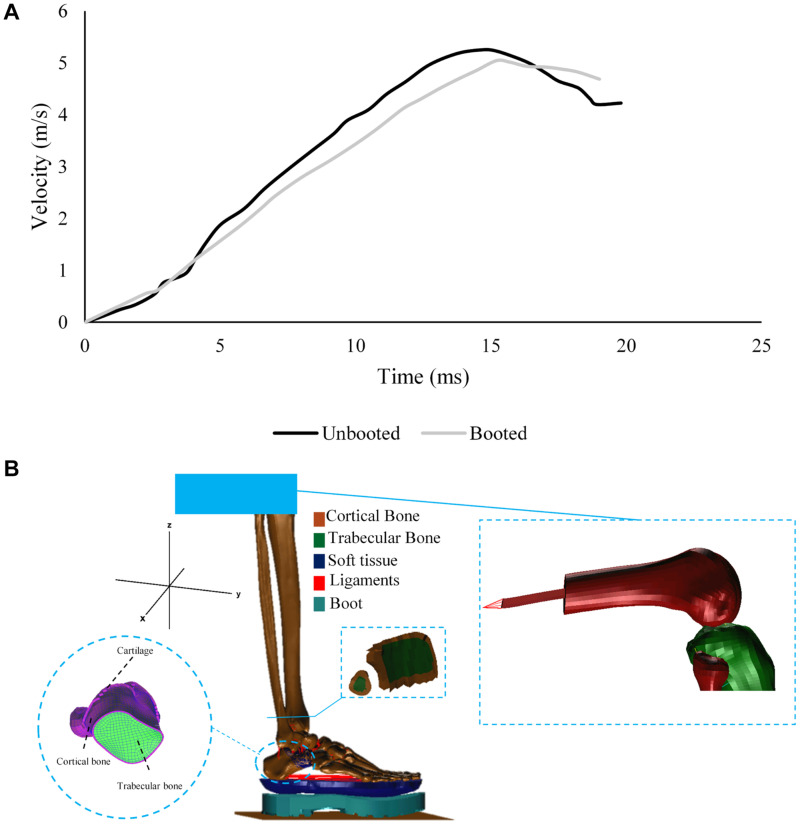
**(A)** Velocity profile of the loading plate during the non-injurious AnUBIS seated lower limb test with and without a combat boot. **(B)** Components of the FE model developed to replicate the experiment on AnUBIS.

The booted and unbooted tests in AnUBIS were simulated with a non-injurious loading case (Figure 1A). The unbooted simulation was used in order to calibrate the FE model prior to testing the model for validity against experiments conducted elsewhere and against the booted AnUBIS experiment.

The distal femur of the cadaveric specimen was created from the available CT scan and included in the FE model (Figure 1B). An intramedullary steel rod was added to the femur and linked to a multiple point constraint with mass of 40 kg to represent the attachment of the femur to the mechanical “hip joint” of AnUBIS. Tibia and fibula were constrained to only move vertically upward, and the femur attachment was unconstrained to rotate in the sagittal plane. A rigid moving floor was added to the model to represent the loading plate and was assigned the acceleration profile measured in the experiment. The floor was constrained to move only vertically upward. Contacts were defined by building master-slave relations between model components. In the case of the contact between cortical and trabecular bone, a standard master-slave contact was used to prevent two surfaces from penetrating each other, define the friction coefficient applied between cartilaginous layers in joints and monitor the contact at the post-processing phase. Additional contacts were defined to represent the interactions between cartilage and other components in the model, as well as between plantar tissue and the rigid floor. The initial model time step was set to 9.5 × 10^–8^ s.

**FIGURE 2:**
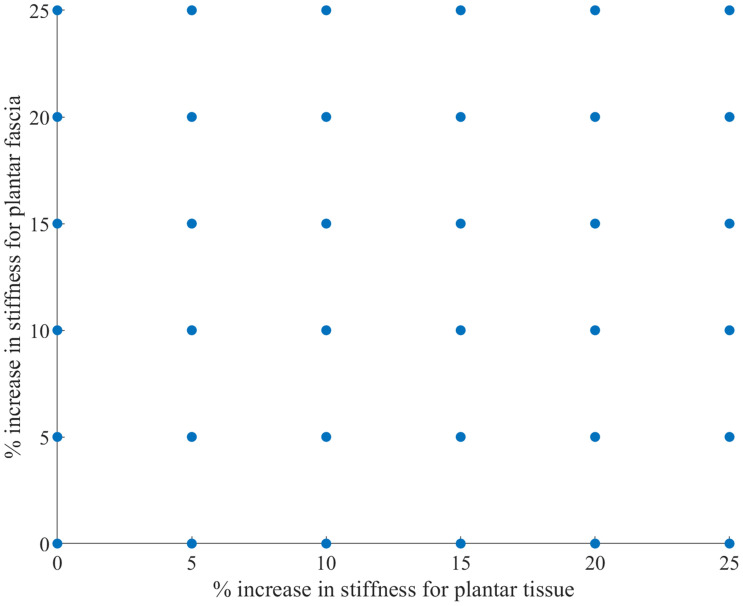
Matrix of combinations of values used to run the sensitivity analysis.

Previous studies (Perry et al., 2014; Grigoriadis et al., 2016) have indicated that there is a significant knowledge gap regarding the material properties for ligamentous and plantar soft tissue. As a result, and in order to calibrate the model, a sensitivity analysis was performed for the spring stiffness of the truss elements representing the plantar fascia and the Mooney-Rivlin constants in the material model for the plantar tissue. The hyperelastic material constants of the plantar tissue were increased incrementally by 5% concurrently with incremental increases of 5% for the stiffness of the plantar fascia(Figure 2). The optimal combination of parameters was obtained after calculating the correlation between the experimental curve and each output curve of the 36 combinations with the CORA plus software (CORAPlus 4.0.4 2017, PDB, Germany). CORAPlus was developed to evaluate the predictive power of computational models through the calculation of the correlation between two time-history signals (Thunert, 2017). It calculates a series of metrics according to ISO Standard 18571 74 that can be used to assess objectively the biofidelity of a computational model. The proposed settings by the developers of CORA were used to define the global and load-case parameters for the analysis. The combination selected had the best agreement on the ratings of magnitude, shape and phase. The initial stiffness selected for plantar fascia was 240  N/mm. The initial Mooney-Rivlin model constants selected for plantar tissue were *C*_*01*_ = 0.1 MPa and *C*_10_ = 1.258 MPa.

### Model Validation – Simulation of Pendulum Test Set Ups

The set of parameters from the sensitivity analysis best matching the experimental data were chosen as inputs of the calibrated model and used for the remainder of the simulations described.

**FIGURE 3:**
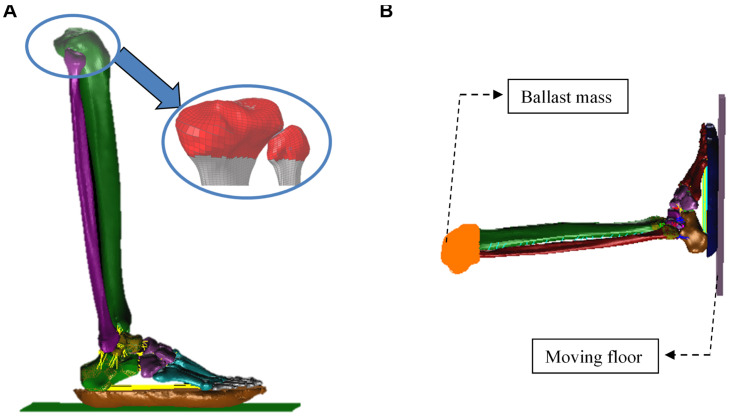
Sagittal views of the finite-element model used to simulate the pendulum tests. The red shell layer models the ballast mass representing the added weight of the load cell and instrumentation, and contraints the tibia and fibula to move in the direction of loading **(A)**. The ballast mass at the proximal tibia was set as 14 or 16 kg depending on the test modality **(B)**.

The boundary conditions of the calibrated model were adjusted to replicate the pendulum impact setup used by Gallenberger et al. (2013) and (Yoganandan et al., 1996). A rigid surface embracing the proximal ends of the tibia and fibula were modeled (Figure 3) and only allowed to move in the direction of pendulum impact. The loading plate was assigned an initial velocity that corresponded to the pendulum impact velocity reported in these studies. The ballast mass at the proximal tibia was set as 14 or 16 kg depending on the test modality (Figure 3). The loading plate was assigned initial velocities between 4 and 6 m/s, depending on the test modality (Table 2), to load the plantar foot.

**TABLE 2:** List of simulations run to replicate the pendulum tests conducted by Yoganandan et al. (1996); Gallenberger et al. (2013) in which PMHS legs in the neutral position were impacted at different impact speeds.

Experimental set-up	Impact velocity (m/s)	Pendulum mass (kg)
Yoganandan et al., 1996	3.3	23
	4.5	
	6.7	
	7.6	
Gallenberger et al., 2013	5	3.3
	5	5.7
	5	12.32
	6	3.3
	6	5.7
	6	12.32

*The combinations of impact velocity and pendulum mass are shown.*

### Modeling of the Combat Boot

**FIGURE 4:**
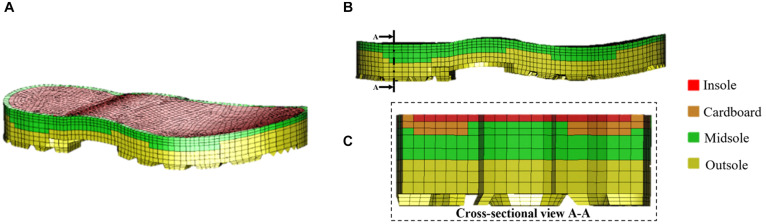
Geometry of the Meindl combat boot showing four material layers **(A)**, lateral side view **(B)** and cross-sectional view (indicated by the cutting plane line A-A) **(C)**.

Model simulations were then run to investigate the utility of the FE model to assess countermeasures. Specifically, the protection offered by the Meindl combat boot was assessed over a range of loading conditions representative of UBB (Gallenberger et al., 2013; Bailey et al., 2015; Yoganandan et al., 2015; Pietsch et al., 2016; Baker et al., 2017). The material parameters used for the combat boot were calculated based on the fit of experimental data obtained in a previous study (Newell et al., 2016). The combat boot is composed of three layers: outsole, midsole and insole (Figure 4).

**FIGURE 5:**
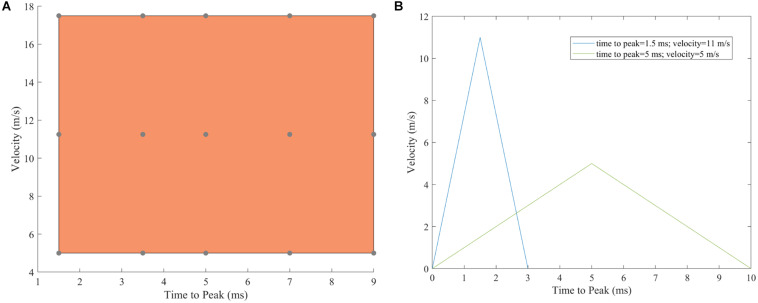
Range of under-body blast simulations run. **(A)** Each point represents the input to the loading floor plate for each simulation. **(B)** Examples of two initial input velocities given to the loading plate that were a triangular pulse.

Under-body blast events are dependent on many environmental (such as soil properties and moisture level), and specimen and mechanical input factors (wide acceleration profile). Due to this complexity and heterogeneity, there is a multitude of acceleration profiles relevant to UBB simulations. Hostetler et al. (2019) developed one of the very few UBB FE models that studied the effects of different loading parameters on injury predictor variables including knee, tibia, and calcaneus loading. The definition of input acceleration profiles was based on UBB environment data reported in previous studies. The geometry of the lower extremity from the Global Human Body Model Consortium (GHBMC) was used and impacted with velocities between 2.5 and 20 m/s, and pulse duration between 2 and 15 ms. The predicted loading data from the model were used to generate a contour plot of lower extremity fracture risk as a function of pulse duration and peak velocity. Multiple loading-plate velocity profiles were also defined and simulated in this study to represent a range of UBB loading (Figure 5). Peak velocity ranged from 5 to 17.5 m/s with time to peak between 1.5 and 9 ms. These values were based on the acceleration profiles reported in the literature (Henderson et al., 2013; Bailey et al., 2015; Yoganandan et al., 2015; Pietsch et al., 2016; Yoganandan and Chirvi, 2016; Baker et al., 2017). The velocity profile was modeled as a triangular shape according to the required combination of peak impact velocity and time to peak.

### Protection Offered by the Combat Boot for the UBB Loading Range

The computational model was used to predict the proximal tibia peak force – as a metric of probability of injury – with and without a combat boot across the UBB loading range. An injury-risk curve which correlates probability of injury with peak axial proximal tibia forces (Chirvi et al., 2017) was used to determine the reduction in risk of calcaneal injury in the booted configuration. The two severity levels considered in the (Chirvi et al., 2017) study, minor and major calcaneal injury risk, follow the application of the Sanders Classification System (Sanders et al., 1993). This classification system categorizes fractures based on fracture displacement and fracture-line localization. In the case of this study, minor calcaneal injuries correspond to a Sanders-I injury with less than 2 mm articular displacement and major calcaneal injuries correspond to Sanders-II injuries with at least one primary fracture line.

### Statistical Analysis

Normal density functions were fit for the predicted values of proximal tibial force for the unbooted and booted configurations to display graphically the predicted tibial force across the loading range. In order to ensure that the datasets for both configurations were sufficiently different, the Student’s *t*-test was used to confirm whether the two datasets were statistically different from each other. This statistical analysis was performed in MATLAB (R2019a, The MathWorks Inc., Natick, MA, United States) using a script that included the ttest2(x,y) function. A two-sample *t*-test was conducted to verify whether the means of normal distributions were equal for a 5% significance level.

## Results

### AnUBIS Tests – Model Calibration

**FIGURE 6:**
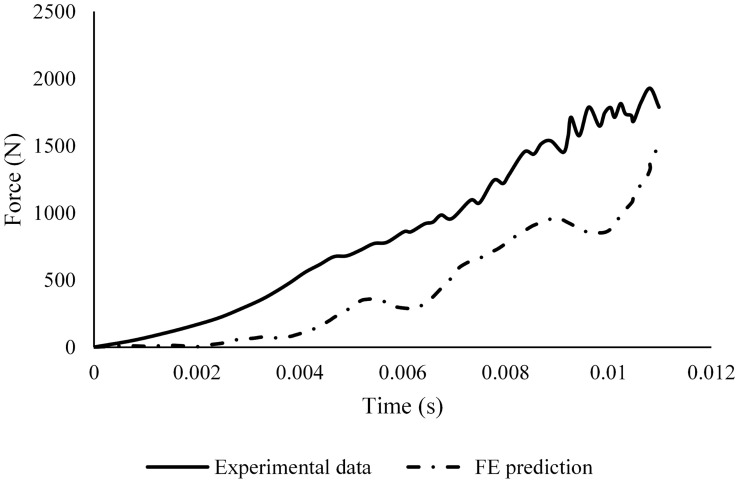
Comparison between the hind-foot force in the unbooted FE model prediction with material properties from literature and the respective signal from the sensor in the AnUBIS experiment.

**FIGURE 7:**
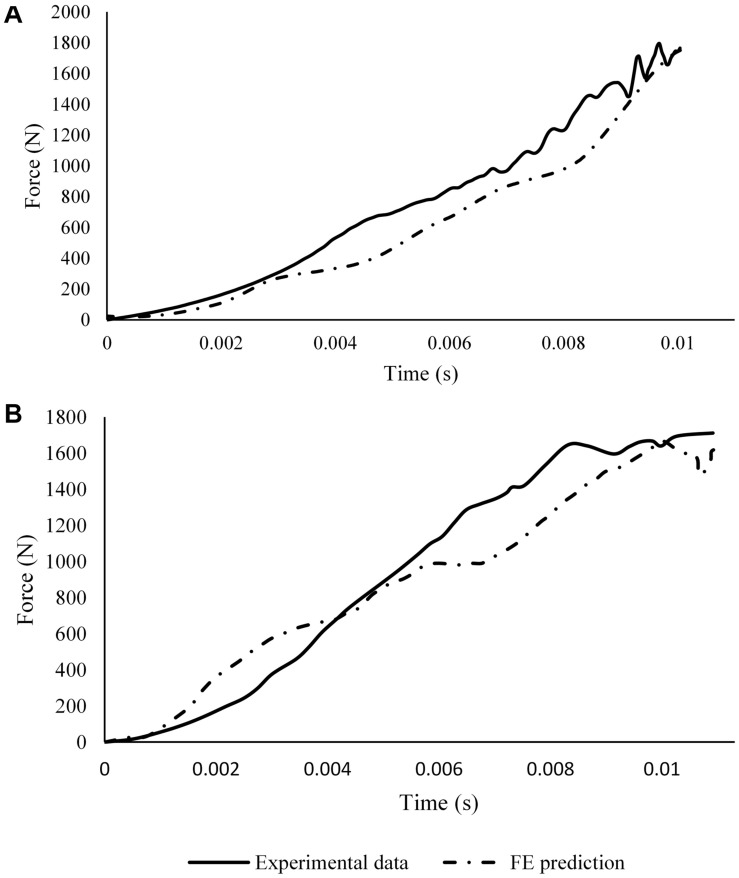
Comparison between calibrated FE prediction of the hindfoot force and sensor data from the experiment **(A)** without and **(B)** with a combat boot.

The hind-foot force of the unbooted FE model in AnUBIS predicted fairly the slope and phase of the experimental hindfoot force signal but only poorly the evolution of its magnitude (Figure 6). The combination of parameter values for plantar fascia and plantar tissue which resulted in the best match between simulation and experiment is shown in Table 3. The CORA metrics of the calibrated FE model (with the input parameters of Table 3) confirm the good fidelity of the FE model in both the unbooted and booted configurations (Table 4). The hind-foot force of the simulations with the calibrated FE model for unbooted and booted configurations is shown against the experimental signals in Figure 7.

**TABLE 3:** Material properties that were shown to be optimal during the calibration of the FE model.

Model component	Material model	Model parameters
Plantar tissue	Quasi-linear viscoelastic	*ρ* = 0.97, *A* = 0.8, *B* = 2.15, *v* = 0.49
Plantar Fascia	Non-linear spring	*k* = 234.6

*A (C_01_) and B (C_10_), Mooney-Rivlin rubber model parameters; v, Poisson’s ratio; ρ, density [g/cm^3^]; k, stiffness [N/mm]. These were used for all subsequent FE models.*

**TABLE 4:** CORA metrics for the outputs of the FE model simulating the experiment in AnUBIS with and without the inclusion of a combat boot.

		CORA rating (according to ISO18571)					
		Magnitude		Phase		Slope	
	Model version	*Metric*	*Grade*	*Metric*	*Grade*	*Metric*	*Grade*
A	Initial Model – No Boot	0.474	Poor	0.608	Fair	0.757	Fair
B	Updated Model – No Boot	0.886	Good	0.854	Good	0.786	Fair
C	Updated Model – With Combat Boot	0.958	Excellent	0.786	Fair	0.970	Excellent

### Model Validation – Pendulum Tests

**FIGURE 8:**
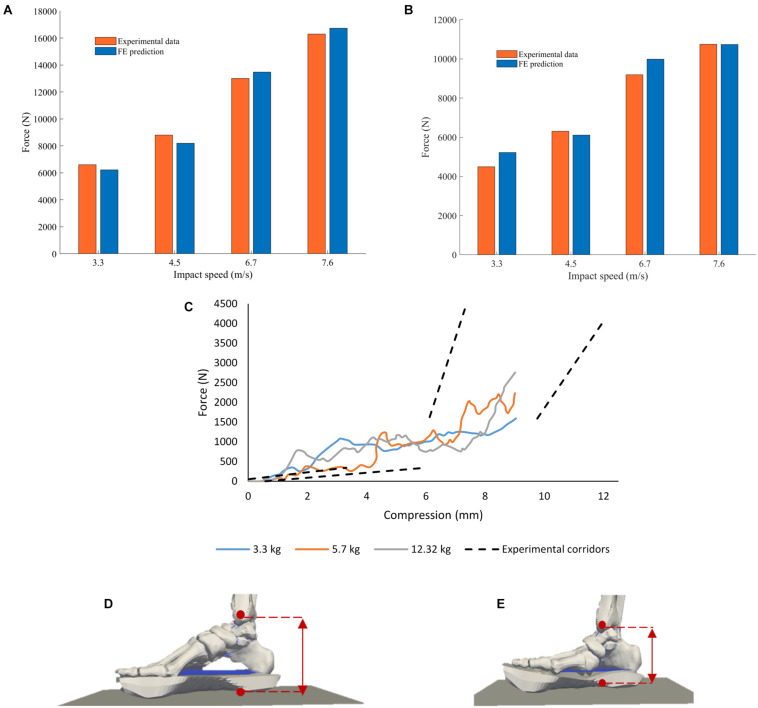
Comparison between peak forces reported by Yoganandan et al. (1996) to those calculated by the computational model at the **(A)** impact floor and **(B)** proximal tibia. **(C)** Proximal Pot Force vs. Foot Compression FE model predictions (solid lines) and experimental corridors (dotted lines) reported by Gallenberger et al. (2013). The simulations were run for pendulum masses of 3.3, 5.7, and 12.32 kg to replicate the experiments conducted. The experimental corridors are 1 standard deviation from the mean response. Foot compression was calculated as the distance between the medial malleolus and the loading plate in the FE model; **(D)** foot in starting unloaded position and **(E)** foot fully compressed in the model with a pendulum mass of 3.3 kg.

The proximal tibial force and the loading-plate force at the heel region predicted by the FE model were compared with the values reported by literature (Yoganandan et al., 1996; Gallenberger et al., 2013). In Yoganandan et al. (1996) the ballast mass was 16 kg and the leg was impacted at velocities between 3.3 and 7.6 m/s (Figure 8); no standard deviations were given. The experiment-FE percentage difference ranged between 0.20 and 16% for the proximal tibia and between 2.6 and 6.9% for the loading plate force.

Foot compression against proximal tibial force from the FE model simulations and the respective pendulum impact experimental corridors with three different pendulum masses reported in Gallenberger et al. (2013) are show in Figure 8C. Foot compression was calculated as the distance between the medial malleolus and the loading plate in the FE model.

### Protection Offered by the Combat Boot Across a Range of UBB Loading

**FIGURE 9:**
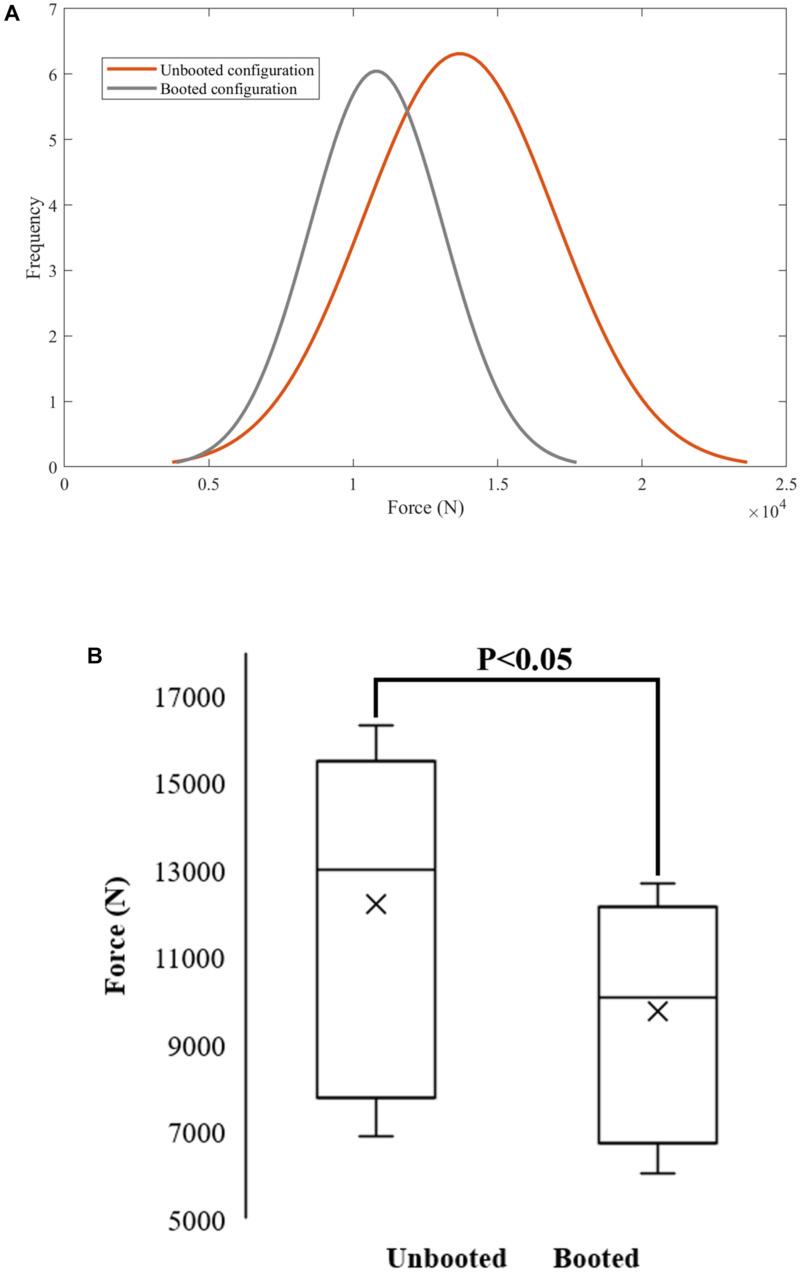
Normal probability density functions for unbooted (μ = 12230.2; σ = 3624.45) and booted (μ = 9764.52; σ = 2497.47) **(A)** were used to quantify the reduction of proximal tibia force across the range of input UBB loads. The null hypothesis was rejected – the means of the normal distributions for the unbooted and booted configurations were not equal for a 5% significance level **(B)**.

The difference between the predicted proximal tibia peak force from the booted to the unbooted configuration was calculated to assess the protection offered by the Meindl combat boot. The difference peaked at the mid-velocity levels beyond which the difference reduced (Table 5). These results are presented using normal density functions in Figure 9. It was found that for combinations with impact velocities above 16 m/s, the risk of injury could not be reduced when the boot was incorporated (i.e., the injury risk remained at 100%). As a result, the predicted reduction in risk of injury to the calcaneus due to the presence of the combat boot for the first 15 combinations is shown in Table 6. It was found that the null hypothesis was rejected.

**TABLE 5:** Difference found between the predicted proximal tibial force from the booted to the unbooted configuration across a range of UBB loading represented by 25 combinations of target peak velocity and time to peak velocity.

Combination	Time to peak velocity (ms)	Peak velocity (m/s)	% Difference in proximal tibia force
1	1.5	5	−12
2	3.5	5	−19
3	5	5	−12
4	7	5	−14
5	9	5	−14
6	1.5	11.25	−24
7	3.5	11.25	−22
8	5	11.25	−21
9	7	11.25	−23
10	9	11.25	−22
11	1.5	17.5	−22
12	3.5	17.5	−22
13	5	17.5	−23
14	7	17.5	−21
15	9	17.5	−25
16	1.5	23.75	−22
17	3.5	23.75	−21
18	5	23.75	−24
19	7	23.75	−19
20	9	23.75	−18
21	1.5	30	−15
22	3.5	30	−19
23	5	30	−19
24	7	30	−17
25	9	30	−18

**TABLE 6:** Predicted reduction in the injury of a calcaneal injury due to incorporation of a Meindl combat boot.

	Change in injury probability between unbooted and booted configurations (%)	
Combination	Calcaneal minor injuries	Calcaneal major Injuries
1	−6	−1
2	−11	−2
3	−6	−2
4	−9	−2
5	−10	−3
6	−37	−46
7	−36	−50
8	−34	−45
9	−30	−54
10	−25	−39
11	−19	−50
12	−18	−47
13	−13	−42
14	−14	−43
15	−12	−42

*The probability was calculated using the injury-risk curve developed by Chirvi et al. (2017).*

## Discussion

An FE model of the leg was developed and calibrated to simulate its response across a range of UBB loading. A sensitivity analysis study was performed to assess how variations in the model’s inputs could improve the agreement between the experimental measures and the predicted force from the numerical model. The spring stiffness assigned to the truss elements representing the plantar fascia and the Mooney-Rivlin constants in the material model for the plantar tissue were selected as the sensitivity analysis inputs. The calibrated model was able to predict the hindfoot force in a traumatic injury simulator set-up and the proximal pot force, loading-plate force, and compression of the foot in a pendulum set-up. This is the first UBB FE model that has been validated against two distinct sets of boundary conditions and against an independent test set up.

The evolution of the curve that relates foot compression with proximal pot force follows the trend that had been reported by Gallenberger et al. (2013) for impact velocities between 4 and 6 m/s. The FE model was developed with a single leg and calibrated using experimental data obtained with one specimen selected to be as representative as possible of military personnel, a 50th percentile male with age at time of death of 35 years. The intention was to develop a patient-specific model so that model calibration and validation can be free of uncertainties due to anatomical variability. Specimens used in the pendulum tests had varying anthropometries and age at time of death, all of which were above 50 years old. The FE model for the pendulum tests over predicts the proximal pot force for the lower values of foot compression, but is within the experimental values reported at the later stages of compression. Age-related tissue degeneration in the specimens used in the pendulum tests may be partly responsible for differences in the biomechanical response of the leg compared to that predicted by the FE model of a 35 years old. Additionally, there are notable differences in the anthropometries, mass properties and age at death between the specimens used in the (Yoganandan et al., 1996; Gallenberger et al., 2013) studies and the specimen geometry used in the computational model. Model calibration was performed through the prediction of load at the forefoot. The pendulum test data used in the validation process includes loading measurements at the proximal tibia. As a result, the divergence observed in the predicted force for lower values of compression can also be attributed to the lack of model calibration for foot compression and plantar flexion. The model performs well when conditions are closer to the UBB injury-inducing patterns that is study is exploring. In fact, at the later stages of compression and higher proximal pot force, the curves are within the experimental corridors reported by Gallenberger et al. (2013).

The combat boot was shown to reduce the proximal pot force between 12 and 14% for velocities below 16 m/s and for times to peak above 5 ms. It was found that for cases with impact velocities above 16 m/s, the combat boot did not reduce sufficiently the predicted proximal tibial force and the probability of sustaining injury was 100%. In order to model the impact conditions of the experiments conducted with the traumatic injury simulator, only the combat boot layers were modeled in this study. While the effect that the upper boot and laces may have on the foot during UBB events is not known, its absence was not expected to affect the predictions of the model because there is very limited flexion of the ankle joint during loading from the upward moving plate and high-rate loading is transmitted axially from the vehicle structure through the combat boot layers and foot. There is a lack of studies that include an assessment of the performance of combat boots in high severity impacts. A recent study by Hampton et al. (2019) included the measurement of peak upper tibial force for an impact with and without McRae combat boots. For an impact with a peak impact velocity of 10 m/s, the upper tibia peak force was found to be 12100 N for the unbooted configuration which would equate to an injury risk probability of 82 and 47% for minor and major calcaneal injuries, respectively. The upper tibia peak force found for the booted configuration was 7540 N which equates to an injury risk probability of 23 and 3% for minor and major calcaneal injuries, respectively. The reported peak tibial force indicates that this model is operating within a sensible range.

The normal density function fit was used as a tool to quantify the reduction of proximal tibia force across the range of input UBB loads. A shift to lower peak axial forces was observed for the booted configuration. The injury-risk reduction was more significant for low severity impacts with times to peak above 5 ms. For the simulations with impact velocity velocities higher than 17.5 m/s there was no reduction in injury risk offered by the combat boot. These findings are consistent with previous studies (Perry et al., 2014; Hampton et al., 2019) that found a notable decrease in peak force and peak strain at lower peak impact velocities and when a combat boot was incorporated. These results are specific to the combat boot type and model used. Although additional simulations need to be run to define the region where current combat boots offer effective protection, this model provides a quantitative measure of the performance of combat boots and allows the incorporation of additional combat boot models.

## Conclusion

A validated FE model of the leg was developed to explore the mechanisms of injury during UBB events in detail and to serve as a tool to characterize the protection offered by blast mitigation systems. This new computational model allows the prediction of injury risk within a range of UBB loading and can be utilized to incorporate other types of mitigation systems.

## Data Availability Statement

The original contributions presented in the study are included in the article/supplementary material, further inquiries can be directed to the corresponding author/s.

## Ethics Statement

Tissue samples were provided by the Imperial College Healthcare NHS Trust Tissue Bank (ICHTB). Other investigators may have received samples from these same tissues. The research was supported by the National Institute for Health Research (NIHR) Biomedical Research Centre based at Imperial College Healthcare NHS Trust and Imperial College London. The views expressed are those of the authors and not necessarily those of the NHS, the NIHR or the Department of Health.

## Author Contributions

AB and SM conceived the study. ER, GG, AB, and SM designed the study. ER, DC, and GG were involved in the preparation of tests, data acquisition, and conducting the tests. ER developed the finite-element models, analyzed the data, and drafted the manuscript. All authors reviewed, edited, and approved the submitted version.

## Conflict of Interest

The authors declare that the research was conducted in the absence of any commercial or financial relationships that could be construed as a potential conflict of interest.

## References

[B1] Bailey A. M. Christopher J. J. Brozoski F. Salzar R. S. (2015). Post mortem human surrogate injury response of the pelvis and lower extremities to simulated underbody blast. *Ann. Biomed. Eng.* 43 1907–1917. 10.1007/s10439-014-1211-5 25503737

[B2] Baker W. A. Chowdhury M. R. Untaroiu C. D. (2018). Validation of a booted finite element model of the WIAMan ATD lower limb in component and whole-body vertical loading impacts with an assessment of the boot influence model on response. *Traffic Injury Prevention* 19 549–554. 10.1080/15389588.2018.1433829 29381394

[B3] Baker W. A. Untaroiu C. D. Crawford D. M. Chowdhury M. R. (2017). Mechanical characterization and finite element implementation of the soft materials used in a novel anthropometric test device for simulating underbody blast loading. *J. Mech. Behav. Biomed. Mater.* 74 358–364. 10.1016/j.jmbbm.2017.06.021 28668593

[B4] Chirvi S. Pintar F. Yoganandan N. Banerjee A. Schlick M. Curry W. (2017). Human foot-ankle injuries and associated risk curves from under body blast loading conditions. *Stapp Car Crash J.* 61 157–173. 29394438 10.4271/2017-22-0006

[B5] Dong L. Zhu F. Jin X. Suresh M. Jiang B. Sevagan G. (2013). Blast effect on the lower extremities and its mitigation: a computational study. *J. Mech. Behav. Biomed. Mater.* 28 111–124. 10.1016/j.jmbbm.2013.07.010 23973770

[B6] Doukas W. C. Hayda R. A. Frisch H. M. Andersen R. C. Mazurek M. T. Ficke J. R. (2013). The Military Extremity Trauma Amputation/Limb Salvage (METALS) study: outcomes of amputation versus limb salvage following major lower-extremity Trauma. *J. Bone Joint Surg. Ser. A* 95 138–145. 10.2106/JBJS.K.00734 23324961

[B7] El-Khoury G. Y. Alliman K. J. Lundberg H. J. Rudert M. J. Brown T. D. Saltzman C. L. (2004). Cartilage thickness in cadaveric ankles: measurement with double-contrast multi-detector row CT arthrography versus MR imaging. *Radiology* 233 768–773. 10.1148/radiol.2333031921 15516604

[B8] Gallenberger K. Yoganandan N. Pintar F. (2013). Biomechanics of foot/ankle trauma with variable energy impacts. *Ann. Adv. Automotive Med.* 57 123–132. PMC3861818 24406952

[B9] Grigoriadis G. Carpanen D. Bull A. M. J. Masouros S. D. (2016). “A finite element model of the foot and ankle for prediction of injury in under−body blast,” in *Proceedings of the 2016 IRCOBI International Research Council on the Biomechanics of Injury*, Vol. 44 Malaga, 457–458.

[B10] Grigoriadis G. Carpanen D. Webster C. E. Ramasamy A. Newell N. Masouros S. D. (2019). Lower limb posture affects the mechanism of injury in under-body blast. *Ann. Biomed. Eng.* 47 306–316. 10.1007/s10439-018-02138-4 30276492 PMC6315016

[B11] Hampton C. E. Kleinberger M. Schlick M. Yoganandan N. Pintar F. A. (2019). Analysis of force mitigation by boots in axial impacts using a lower leg finite element model. *Stapp Car Crash J.* 63 267–289. 32311060 10.4271/2019-22-0011

[B12] Hansen U. Zioupos P. Simpson R. Currey J. D. Hynd D. (2008). The effect of strain rate on the mechanical properties of human cortical bone. *J. Biomech. Eng.* 130:011011. 10.1115/1.2838032 18298187

[B13] Henderson K. A. Bailey A. M. Christopher J. J. Brozoski F. Salzar R. S. (2013). “Biomechanical response of the lower leg under high rate loading,” in *Proceedings of the 2013 IRCOBI International Research Council on the Biomechanics of Injury, September*, Gothenburg, 145–157.

[B14] Hostetler Z. S. Aira J. Stitzel J. D. Gayzik F. S. (2019). “A computational study of the biomechanical response of the human lower extremity subjected to high rate vertical accelerative loading,” in *IRCOBI Conference*, Florence, 662–673.

[B15] Iwamoto M. Miki K. Tanaka E. (2005). Ankle skeletal injury predictions using anisotropic inelastic constitutive model of cortical bone taking into account damage evolution. *Stapp Car Crash J.* 49 133–156. 17096272 10.4271/2005-22-0007

[B16] Kitaoka H. B. Luo Z. P. Growney E. S. Berglund L. J. An K. N. (1994). Material properties of the plantar aponeurosis. *Foot Ankle Int.* 15 557–560. 10.1177/107110079401501007 7834064

[B17] Masouros S. D. Newell N. Ramasamy A. Bonner T. J. West A. T. H. Hill A. M. (2013). Design of a traumatic injury simulator for assessing lower limb response to high loading rates. *Ann. Biomed. Eng.* 41 1957–1967. 10.1007/s10439-013-0814-6 23612913

[B18] McElhaney J. H. (1966). Dynamic response of bone and muscle tissue. *J. Appl. Physiol.* 21 1231–1236. 10.1152/jappl.1966.21.4.1231 5916656

[B19] Millington S. A. Grabner M. Wozelka R. Anderson D. D. Hurwitz S. R. Crandall J. R. (2007). Quantification of ankle articular cartilage topography and thickness using a high resolution stereophotography system. *Osteoarthritis Cartilage* 15 205–211. 10.1016/j.joca.2006.07.008 16949841

[B20] Newell N. Salzar R. Bull A. M. J. Masouros S. D. (2016). A validated numerical model of a lower limb surrogate to investigate injuries caused by under-vehicle explosions. *J. Biomech.* 49 710–717. 10.1016/j.jbiomech.2016.02.007 26924659

[B21] Pal G. P. Routal R. V. (1998). Architecture of the cancellous bone of the human talus. *Anat. Rec.* 252 185–193. 10.1002/(sici)1097-0185(199810)252:2<185::aid-ar4>3.0.co;2-2 9776073

[B22] Perry B. J. Gabler L. Bailey A. Henderson K. Brozoski F. Salzar R. S. (2014). “Lower extremity characterization and injury mitigation,” in *IRCOBI Conference Proceedings – International Research Council on the Biomechanics of Injury*, Berlin, 186–197.

[B23] Pietsch H. A. Bosch K. E. Weyland D. R. Spratley E. M. Henderson K. A. Salzar R. S. (2016). Evaluation of WIAMan technology demonstrator biofidelity relative to sub-injurious PMHS response in simulated under-body blast events. *Stapp Car Crash J.* 60 199–246. 27871099 10.4271/2016-22-0009

[B24] Ramasamy A. Hill A. M. Masouros S. Gibb I. Bull A. M. J. Clasper J. C. (2011). Blast-related fracture patterns: a forensic biomechanical approach. *J. R. Soc. Interface* 8 689–698. 10.1098/rsif.2010.0476 21123255 PMC3061094

[B25] Ramasamy M. A. Hill A. M. Masouros S. Bull A. M. J. Gibb L. C. I. Phillip L. C. R. (2013). Outcomes of IED foot and ankle blast injuries. *J. Bone Joint Surg. Ser. A* 95 1–7. 10.2106/JBJS.K.01666 23467873

[B26] Sabry F. F. Ebraheim N. A. Mehalik J. N. Rezcallah A. T. (2000). Internal architecture of the calcaneus: implications for calcaneus fractures. *Foot Ankle Int.* 21 114–118. 10.1177/107110070002100204 10694022

[B27] Sanders R. Fortin P. DiPasquale T. Walling A. (1993). Operative treatment in 120 displaced intraarticular calcaneal fractures: results using a prognostic computed tomography scan classification. *Clin. Orthopaedics Related Res.* 290, 87–95. 10.1097/00003086-199305000-00012 8472475

[B28] Shin J. Yue N. Untaroiu C. D. (2012). A finite element model of the foot and ankle for automotive impact applications. *Ann. Biomed. Eng.* 40 2519–2531. 10.1007/s10439-012-0607-3 22695987

[B29] Sommer E. Olaf J. M. (1994). Determination of mechanical properties of microcomponents. *Materialpruefung/Mater. Testing* 36 124–128.

[B30] Thunert C. (2017). *CORAplus Release 4.0.4 Users’ Manual* (Issue May). Partnership for Dummy Technology Biomechanics.

[B31] Yoganandan N. Chirvi S. (2016). Foot – ankle fractures and injury probability curves from post-mortem human surrogate tests. *Ann. Biomed. Eng.* 44 2937–2947. 10.1007/s10439-016-1598-2 27052746

[B32] Yoganandan N. Pintar F. A. Boynton M. Begeman P. Prasad P. (1996). *Dynamic Axial Tolerance of the Human Foot-Ankle Complex.* Warrendale: SAE International.

[B33] Yoganandan N. Pintar F. A. Schlick M. Humm J. R. Voo L. Merkle A. (2015). Vertical accelerator device to apply loads simulating blast environments in the military to human surrogates. *J. Biomech.* 48 3534–3538. 10.1016/j.jbiomech.2015.06.008 26159057

